# Properties Related to the HLB Value of Hybrid Thermoelectric Nanofluids at Different Temperatures

**DOI:** 10.3390/polym16040509

**Published:** 2024-02-13

**Authors:** Rong-Tsu Wang, Jung-Chang Wang

**Affiliations:** 1 Department and Graduate Institute of Information Management, Yu Da University of Science and Technology (YDU), Miaoli County 36143, Taiwan; rtwang@ydu.edu.tw; 2 Department of Marine Engineering (DME), National Taiwan Ocean University (NTOU), Keelung 202301, Taiwan

**Keywords:** hybrid nanofluid, polymeric emulsifiers, HLB value, zeta potential, thermoelectric

## Abstract

This article aims to explore the impact of HLB (Hydrophile-Lipophile Balance) values on two key properties, namely the thermoelectric conductivities and the stability of the suspension, of a hybrid nanofluid composed of TiO_2_ and CuO nanoparticles. The present study employed a two-step synthesis method to prepare the polymeric nanofluid, which meant that the nanoparticles were mixed with the base fluid using an ultrasonic oscillator, which was easier and cheaper than the one-step synthesis method. To ensure that the nanoparticles remain evenly dispersed in the base fluid, two distinct polymer-emulsifier combinations with different HLB values were employed as the dispersing agents. The first pair of polymeric emulsifiers consisted of Span#20 and Tween#20, and the second pair was Span#80 and Tween#80 composed to four HLB values of 12, 13, 14, and 15. The experiment measured the properties of the nanofluid, including the particle size, Zeta potential, and thermoelectric conductivities at different temperatures from 20 °C to 50 °C. The experimental outcomes indicated that an HLB value of 13 was the best for the two sets of polymeric emulsifiers tested. This value corresponded to the most reduced particle size, measured at 170 nm, alongside the most elevated Zeta potential, recorded at −30 mV. Additionally, this HLB value was associated with the peak thermoelectric conductivity, which was 1.46 W/m∙K. This suggests that there may be some variation in the best HLB value depending on the type of polymeric emulsifiers and the temperature of the hybrid nanofluid.

## 1. Introduction

Industrial heat dissipation is a challenging problem in the modern era of technological development. Conventional methods such as air cooling, water cooling, oil cooling, heat pipes [1,2,3,4,5], or a vapor chamber [6,7,8,9,10] are not enough to handle the large amount of heat generated by industrial processes. Therefore, nanofluid [11,12,13], a fluid with suspended nanoparticles at the nanoscale, has emerged as a promising solution for enhancing thermal conductivity. Nanofluid consists of a base fluid and nanoparticles, which can be selected from various materials such as oils, ethylene glycol, water, metals, metal oxides, metal carbides, or metal nitrides. A hybrid nanofluid, which is based on a single nanofluid, has even better thermal conductivity than a single nanofluid. The thermal conductivity of a hybrid nanofluid depends on many factors, such as the preparation process, the type, shape, size, and stability of the nanoparticles, and so on. Compared to single nanofluids, hybrid nanofluids have superior thermophysical properties and more flexibility in adjusting their characteristics. By changing the kind and proportion of nanoparticles, hybrid nanofluids can achieve different performance levels. Single nanofluids can also alter their properties by modifying various factors such as nanoparticle size, shape, concentration, temperature, pH, surfactant, and ultrasonic time. However, hybrid nanofluids have more parameters to consider, such as the relative diameter and the mixing ratio of nanoparticles [14,15,16,17]. According to Singh et al. [18], in Al_2_O_3_ nanofluids, the lower the surface potential, the easier it is to cause particle aggregation and precipitation. The addition of polymeric surfactants affects the isoelectric point (IEP) of aluminum oxide, which can be moved away from the IEP of the nanofluid by adjusting the pH value of the solution. Many researchers have investigated the thermophysical and rheological properties of hybrid nanofluids. The above properties are also influenced by the mixing ratio of nanoparticles, which is a key factor that needs to be considered. Kanthimathi et al. [19] conducted a study to investigate the effects of single-component and hybrid nanofluids on the heat transfer process and the thermophysical properties of different base fluids. The study also presented a case study of SiC nanoparticles suspended in a mixture of ethylene glycol and water. Researchers use both experimental and computational methods to study these properties [20].

Hemmat and Esfandeh [21] proposed a new generation of hybrid nanofluids that were composed of TiO_2_ and Al_2_O_3_ nanoparticles dispersed in a synthetic oil as the base fluid. The study also evaluated the thermal conductivity, viscosity, and density of the nanofluids at different temperatures and concentrations. Xian et al. [22] conducted a study comparing the effects of different polymers of surfactants and ultrasonic treatment times on the stability and thermal properties of hybrid nanofluids through the deposition phenomena of nano-powder, analysis of zeta potential, and absorption performance. The results showed that when the polymer of Cetrimonium bromide (CTAB) was used as a dispersant, the stability of the nanofluid could be maintained for at least 40 days, and the thermal conductivity could be increased by 23.74% at a temperature of 60 °C. Additionally, at the same temperature and concentration, the thermal conductivity of the hybrid nanofluids was higher than those of the single nanofluids. Wanatasanappan et al. [23] conducted a study on the effect of the powder ratio of an Al_2_O_3_/CuO hybrid nanofluid on thermal physics. The hybrid nanofluid was prepared using a two-step method, and the ultrasonic oscillation time was 90 min. The surfactant used was LAS (linear alkyl benzene sulfonate), and the ratio of Al_2_O_3_ to CuO nanometer powders was 20:80, 40:60, 50:50, and 60:40. The results showed that the best dispersion stability was achieved when the ratio of Al_2_O_3_ to CuO nanometer powders was 50:50, because the surfactant LAS has good hydrophobicity, which increases the dispersion stability. The best thermal conductivity value was achieved when the ratio of Al_2_O_3_ to CuO nanometer powders was 60:40. Zufar et al. [24] compared the thermal properties of two types of hybrid nanofluids, each containing 0.1 wt% of CuO nanoparticles and either Al_2_O_3_ or SiO_2_ nanoparticles, in a pulsating heat pipe under different conditions of heat and fill ratio. They observed that the Al_2_O_3_-CuO nanofluid had a higher thermal conductivity than the SiO_2_-CuO nanofluid at the same concentration and that both nanofluids reduced the thermal resistance of the heat pipe significantly compared to water. The reduction was 34% for the Al_2_O_3_-CuO nanofluid and 57% for the SiO_2_-CuO nanofluid. Sandhya et al. [25] studied two types of nanofluids: one with only GNP nanoparticles and another with both GNP and CNC nanoparticles. They explained how the nanofluids were made and how they behaved in terms of heat and other physical aspects. They utilized different methods to look at the nanoparticles and the nanofluids and employed a standard test to measure how well the nanofluids can transfer heat at different temperatures from 20 °C to 50 °C. There were different amounts of nanoparticles in the nanofluids, from 0.01 vol.% to 0.2 vol.%. For the single-GNP nanofluid, the thermal conductivity values at room temperature range from 0.366 W/(m·K) to 0.441 W/(m·K), and for the hybrid nanofluid, the thermal conductivity values range from 0.501 W/(m·K) to 0.551 W/(m·K). Finally, based on the results, it can be determined that the thermal performance of the selected nanoparticles is favorable, and the hybrid nanofluid is an acceptable alternative to conventional/water-based fluids in operating systems. Abdullah et al. [26] discussed the effects of nanoparticles on the density, viscosity, thermal conductivity, and specific heat capacity of hybrid nanofluids (water/ethylene glycol-based) at different volume fractions. Two types of nanoparticles with different shapes and sizes were used: alumina (Al_2_O_3_) and zinc oxide (ZnO). The experimental results showed that the density, viscosity, and thermal conductivity of the hybrid nanofluids increased with the increase in nanoparticle concentration and temperature, while the specific heat capacity decreased. The properties of the hybrid nanofluids were also influenced by the mixing ratio of nanoparticles. When the Al_2_O_3_/ZnO mixing ratio changed from 1:1 to 1:3, the density, viscosity, and thermal conductivity of the hybrid nanofluids reached the maximum values, while the specific heat capacity reached the minimum value. The article also used some mathematical models to predict the properties of the hybrid nanofluids and compared them with the experimental data.

The production of nanofluids can be done by either the one-step method or the two-step method, as explained by Sajid et al. [27]. The one-step method combines the synthesis of nano-powder and the dispersion of nanofluids in a single process. It uses techniques such as metal vapor condensation, microwave radiation, and plasma arc to create the nano-powder in a base fluid. This method has the advantages of high stability and uniformity, but it is costly and difficult to scale up. On the other hand, the two-step method separates the synthesis of the nano-powder from the dispersion of nanofluids. It involves adding pre-made nano-powder to a base fluid and then applying ultrasonic vibration to break up the agglomerates. This method is more suitable for mass production and is cost-effective, but it has the drawbacks of complex preparation and aggregation tendency. In summary, the one-step method is advantageous for its high stability and uniformity, while the two-step method is more suitable for mass production and cost-effectiveness [28,29,30]. However, the two-step method requires complex preparation and has an aggregation tendency. Nanofluids have potential applications in various heat transfer systems, such as cooling liquids for refrigeration, heat exchangers, electronic cooling, and so on. Muneeshwaran et al. [31] reviewed the use of hybrid nanofluids in different heat transfer devices, such as heat exchangers, radiators, heat pipes, solar photovoltaic modules, refrigeration and air conditioning systems, and energy storage, among others. They also summarized the fluid properties of different types of hybrid nanofluids and found that the operating temperature affects the thermal performance and flow behavior of hybrid nanofluids. They concluded that the challenges of stability and durability need to be addressed before hybrid nanofluids can be widely used in industry. The objective of the present study was to determine the best thermoelectric conductivities and suspension stability of a hybrid nanofluid. The researchers conducted a detailed measurement of the Hydrophilic-Lipophilic Balance (HLB) [32] value of the nanofluid to determine the best HLB value for the TiO_2_/CuO hybrid nanofluid. In the realm of practical applications, the HLB value plays a pivotal role in the selection of appropriate polymeric surfactants for processes such as emulsification, solubilization, and cleansing. This ensures peak efficacy by aligning with the specific polarity and thermal conditions of the target oil or grime. As a fundamental factor in concocting emulsions and similar mixtures, the HLB value gauges the affinity of a polymeric surfactant towards water (Hydrophilic) or oil (Lipophilic), contributing to the stabilization of suspensions. By assessing the particle size and Zeta potential against the thermoelectric conductivity at varying temperatures, one can pinpoint the most effective HLB value. The insights gleaned from this research are instrumental in enhancing the thermoelectric conductivities and the enduring stability of hybrid nanofluid suspensions.

## 2. Materials and Methodology

**Figure 1:**
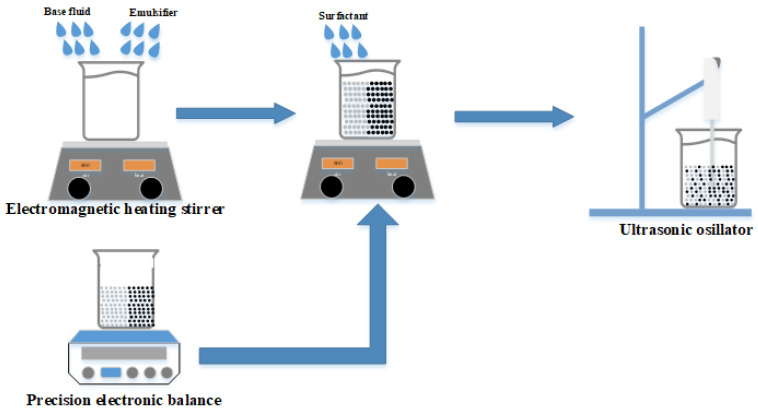
Experiment framework of the hybrid nanofluid.

This section describes the experimental setup and procedures for the preparation and production of hybrid nanofluids and the fabrication of thermoelectric nanofluids. It also discusses the methods for measuring the thermal conductivity, viscosity, and density of the thermoelectric hybrid nanofluids with different hydrophilic-lipophilic balance (HLB) values of polymeric emulsifiers under varying powder concentrations and temperature conditions. Figure 1 displays the methodology of the experiment, which was designed to synthesize a TiO_2_/CuO hybrid nanofluid. To formulate four distinct nanofluids, each with varying HLB values, the base fluid was combined with a pair of polymeric emulsifiers. Throughout the course of this study, the proportions of the emulsifiers, the dispersant, and the ratio of the powders, as well as the total concentration, were meticulously maintained at a steady 1 wt.%.

The suite of instruments utilized for synthesizing hybrid nanofluids and assessing their characteristics included the following. The Shimadzu Corporation’s ATY224 precision electronic balance, originating from Japan, boasts the ability to measure masses up to 220 g with a remarkable precision of 0.1 mg. This device is specifically calibrated for the precise measurement of two distinct nano-powders and a polymeric emulsifier. The PC-420D electromagnetic heating stirrer, crafted by Corning in the USA, is engineered for the homogenization of polymeric emulsifiers, base fluids, and nano particle-infused solutions. It features a rotational speed control spectrum ranging from 60 rpm to 1150 rpm. The HOYU Ultrasonic 250, a creation of Hoyu Technology Co., Ltd. in Taipei, Taiwan, serves as an apparatus tailored for the shattering and even distribution of nanoparticles within nanofluids. It possesses a processing volume span of 0.2 mL to 400 mL and provides versatile operation modes including manual, automatic, continuous, and timed. The KD2 Thermal Properties Analyzer, a product of Decagon Devices in the USA, is capable of gauging thermal conductivity within the bounds of 0.02 to 2 W/m∙K, accompanied by an error margin of ±5%. This instrument employs the transient hot wire method principle, facilitating swift and effortless measurements. Lastly, the Particle Size and Zeta Potential Analyzer, employed for the quantification of particle sizes in concentrations from 0.1 PPM to 40 wt.%, is executed by the Swiss enterprise Malvern utilizing the back-scattering approach.

The preparation of the hybrid nanofluid was carried out through a bifurcated (two-step) synthesis method. Initially, four distinct polymeric emulsifiers were utilized. Subsequently, these emulsifiers were segregated into two sets including Span#20 and Tween#20 (T/S 20), as well as Span#80 and Tween#80 (T/S 80). This division was instrumental in establishing various HLB indices. These indices correspond to diverse fatty acids, which are classified based on their affinity towards lipids or water, known as lipophilic or hydrophilic, respectively. The characteristics of these polymeric emulsifiers are detailed in Table 1. The HLB is determined by the molecular weights of the hydrophilic (water-attracting) and lipophilic (oil-attracting) segments of a surfactant molecule. The HLB value is crucial as it forecasts the surfactant’s performance and characteristics in different settings. An HLB value below 10 signifies that the surfactant dissolves in lipids (is water-insoluble), making it ideal for formulating water-in-oil (W/O) emulsions. Conversely, an HLB value above 10 indicates that the surfactant is water-soluble (lipid-insoluble), thus being well-suited for oil-in-water (O/W) emulsions. To derive HLB indices of 12, 13, 14, and 15, the requisite mass of the polymeric emulsifier is ascertainable through Equation (1) [32]. HLB_AB_ is the HLB value of the solution obtained by mixing polymeric emulsifier A and polymeric emulsifier B. HLB_A_ is the HLB value of polymeric emulsifier A. HLB_B_ is the HLB value of polymeric emulsifier B. W_A_ (g) is the weight of polymeric emulsifier A with a unit of gram. W_B_ (g) is the weight of emulsifier B.

(1) $${HLB}_{AB}=\frac{\left({HLB}_{A}\times W_{A}\right)+\left({HLB}_{B}\times W_{B}\right)}{\left(W_{A}+W_{B}\right)}$$

The primary focus of our initial research phase was to determine the best hydrophilic-lipophilic balance (HLB) value for the Tween and Span mixture in our hybrid nanofluid. For our study, we chose HLB values ranging from 12 to 15 for the Tween and Span 20, as well as the Tween and Span 80 combinations. This range was selected to aid in identifying the best HLB value for each set and to streamline their comparative analysis. Preliminary findings suggest that the polymeric surfactant’s HLB value markedly affects nanofluid properties such as particle size and zeta potential within the temperature range of 20 °C to 50 °C. The ideal HLB value is determined by the desired particle size, zeta potential, and other critical characteristics. In our experiments, an HLB value of 12 led to the minimum particle size, while values of 13 and 14 produced greater zeta potentials, indicating improved nanofluid stability. The present research involved measuring thermal conductivity and analyzing zeta potential and particle size from 20 °C to 50 °C. We utilized two groups of polymer emulsifiers with HLBs of 12, 13, 14, and 15, created by mixing Span#20 with Tween#20 (T/S 20) and Span#80 with Tween#80 (T/S 80). These emulsifiers were combined with deionized water at a concentration of 1 wt.% and stirred using an electromagnetic heating stirrer at 800 rpm for 30 min. Following this, nano-powders were added in equal proportions at a concentration of 1 wt.% to the surfactant mixture. Stirring continued at 800 rpm for another 30 min. The resulting hybrid nanofluid underwent a 2-h ultrasonic oscillation to disperse the nano-powders and boost the nanofluid’s stability.

## 3. Results

The HLB value has been determined as the critical factor affecting the TiO_2_/CuO preparation process, according to established guidelines. We have opted for a 1.0 wt.% concentration of TiO_2_/CuO for this investigation. Additionally, two different polymeric emulsifier sets have been selected. To commence our evaluation, we performed tests measuring particle size, zeta potential, and thermal conductivity on both polymeric emulsifier sets, which have HLB values ranging from 12 to 15. The experimental trials were carried out over a period of 10 consecutive days to determine the best HLB value that would be most suitable for our specific application. This series of tests aimed to build upon the foundational research presented in prior studies, specifically those documented in references [33,34]. The methodology was meticulously designed to align with these earlier works, ensuring consistency and comparability in the results obtained. To investigate the effect of temperature changes on nanoparticle size in nanofluids, we conducted seven measurements over ten days. This approach aimed to identify the best HLB sample from each category. We discovered that nanoparticle size influences the thermal conductivity of nanofluids. Moreover, nanoscale powders can aggregate due to interparticle forces, compromising the fluid’s stability and suspension properties.

Table 2 and Table 3 illustrate the relationships between particle size, temperature variations, and the HLB for two polymetric surfactant combinations involving Span#20/Tween#20 (T/S 20) and Span#80/Tween#80 (T/S 80). As the temperature increases, both particle size and lattice constants tend to grow. This expansion leads to a decrease in the surface tension of the nanocrystals dispersed in the nanofluid, which in turn encourages the particles to cluster together. The results indicated that nanofluids formulated with T/S 20 consistently produced the smallest particle sizes at four distinct temperatures, which was reflected in an HLB value of 13. This suggests a more hydrophilic nature, favoring stability and dispersion in the nanofluid. On the other hand, nanofluids containing T/S 80 also achieved their smallest particle sizes at these temperatures, but with a slightly lower HLB value of 12. The reduced HLB value pointed to increased hydrophobicity, which implied that the polymeric emulsifier’s ability to form a stable colloidal system was influenced by the hydrophilic properties of the base fluid. The base fluid was deionized water, which tends to encourage the aggregation of nanoparticles due to its inherent hydrophilicity in the present study. The findings underscore the delicate balance between surfactant composition and environmental factors in determining the behavior of nanofluids.

**Figure 2:**
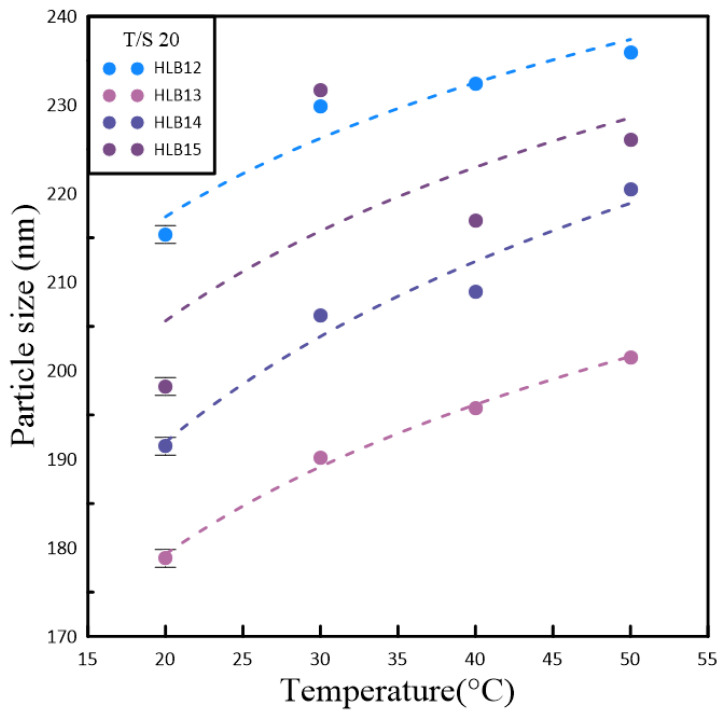
The temperature variation trend chart of suspended particle size in nanofluids based on the HLB value adjustment of Span#20 and Tween#20.

**Figure 3:**
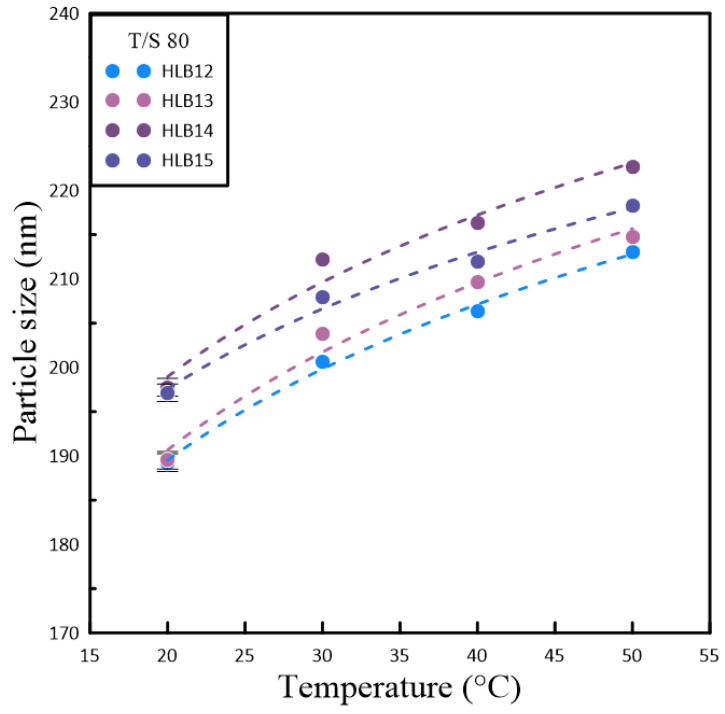
The temperature variation trend chart of suspended particle size in nanofluids based on the HLB value adjustment of Span#80 and Tween#80.

Figure 2 and Figure 3 present a detailed examination of the interplay between nanoparticle size within a solution and temperature, utilizing a quartet of unique polymeric emulsifiers. The data depicted in these figures suggest a consistent and direct relationship. As the temperature of the solution rises, there is a corresponding increase in nanoparticle size. This phenomenon is further underscored by the study’s findings on the impact of temperature on both the dimensions of the particles and their zeta potential, which influences stability for the measure of surface charge. The observed trend of a positive correlation indicated that elevated temperatures lead to an uptick in nanoparticle size, which can be attributed to a faster rate of particle aggregation under such thermal conditions. The rise in temperature boosts the kinetic energy of the particles in the nanofluid, which, in turn, affects how they interact with one another. However, the research team encountered significant challenges in accurately measuring the thermal conductivity of the samples. Technical difficulties with the KD2 thermal conductivity apparatus arose during the experimental process, leading to disruptions in the continuous monitoring of the T/S 80 sample’s thermal behavior over time. Consequently, the data collected over a span of five days may not fully reflect the true thermal conductivity values, as the malfunctioning equipment could have introduced errors. Despite these technical obstacles, the study persevered with the measurements, albeit with the understanding that the resultant data might be compromised to some extent due to the equipment issues. This acknowledgment serves as a reminder of the importance of reliable instrumentation in conducting precise scientific research. A thorough dissection of the information provided by these graphs enabled the identification of the most stable emulsifier based on the HLB values. Notably, the polymeric emulsifier sample containing T/S 80 demonstrated remarkable stability compared to its counterparts.

**Figure 4:**
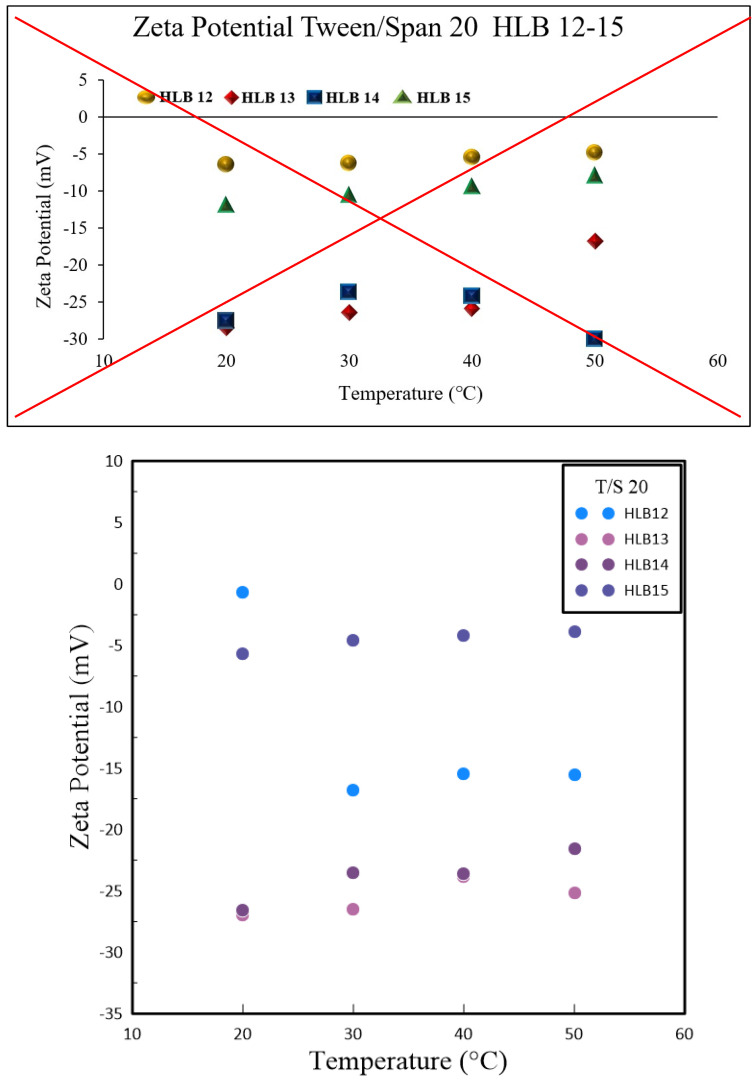
Graphical relationship between Zeta Potential and varying HBL values using 1.0 wt.% Tween and Span #20.

**Figure 5:**
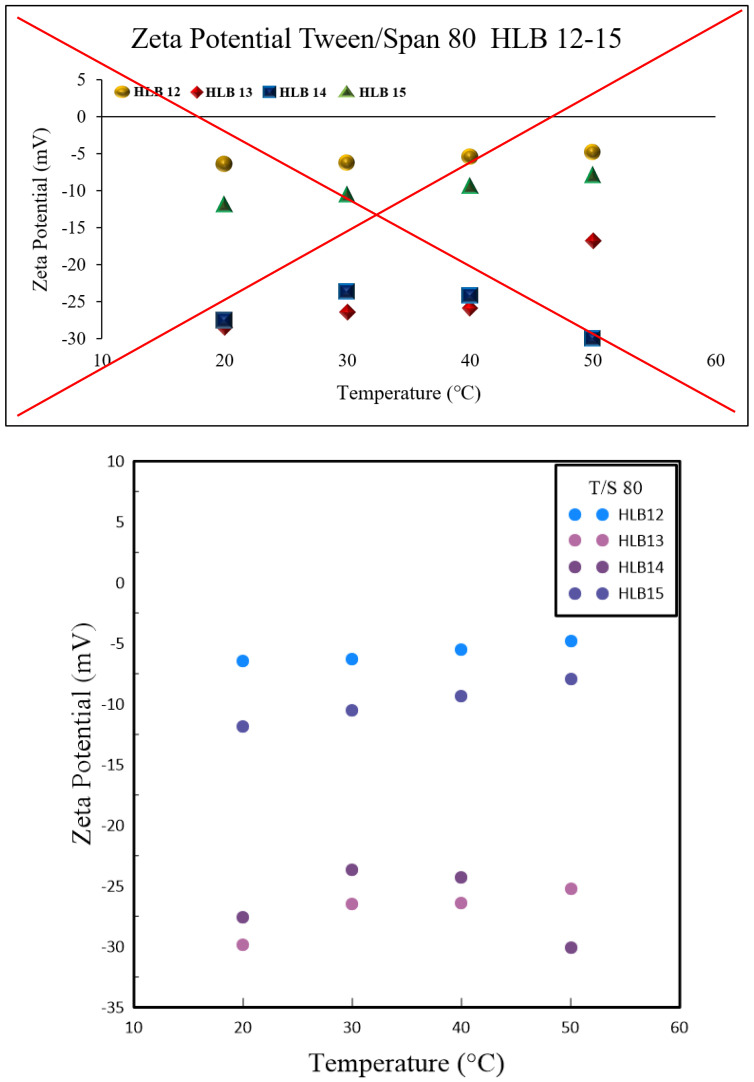
Graphical relationship between Zeta Potential and varying HBL values using 1.0 wt.% Tween and Span #80.

In nanoparticle solutions, ions form a double layer around the particles to stabilize their surface charge. These nanoparticles undergo Brownian motion, displaying erratic movements and carrying the double layer along. This interaction results in electrophoretic motion and the formation of a slipping plane where the double layer meets the nanoparticle surface, a phenomenon referred to as the Zeta potential. Experimental observations have shown that irrespective of the preparation technique, HLB values of 13 and 14 yield better results than those of 12 and 15, as demonstrated in Figure 4 and Figure 5. Specifically, nanofluids with HLB values of 13 and 14 exhibited a higher Zeta potential when compared to those with an HLB value of 12. The Zeta potential is a measure of the electrostatic interactions among particles and is a crucial metric for assessing the stability of nanoparticles within a dispersion. Nanoparticles with higher absolute Zeta potential values are more stable due to increased repulsive forces that reduce the likelihood of aggregation. Consequently, the elevated Zeta potential associated with HLB values of 13 and 14 indicates enhanced stability for these nanofluids.

These results underscore the significance of selecting the appropriate surfactant and fine-tuning the HLB value when formulating nanofluids. A reduction in particle size was observed at an HLB value of 12, whereas HLB values of 13 and 14 hinted at the possibility of improved nanofluid stability, as evidenced by the higher Zeta potential. This research shed light on the intricate relationship between polymeric surfactant composition, HLB value, and temperature in dictating the properties of nanofluids. It emphasizes the need for a comprehensive strategy to tailor nanofluid formulations for specific uses. Colloids with higher absolute Zeta potential values tend to preserve electrical balance and are less prone to coagulate with colloids exhibiting lower Zeta potential. The experimental data confirmed that, for both methods of preparation, HLB values of 13 and 14 were preferable over 12 and 15. This superiority is linked to the disproportionate hydrophobic-hydrophilic ratio in Span and Tween at HLB values of 12 and 15, which leads to a thinner colloidal layer around the nanoparticles and a diminished Zeta potential. Elevated temperatures contribute to nanoparticle clustering, which reduces the solution’s surface area and the count of slipping planes, thereby influencing the Zeta potential.

**Figure 6:**
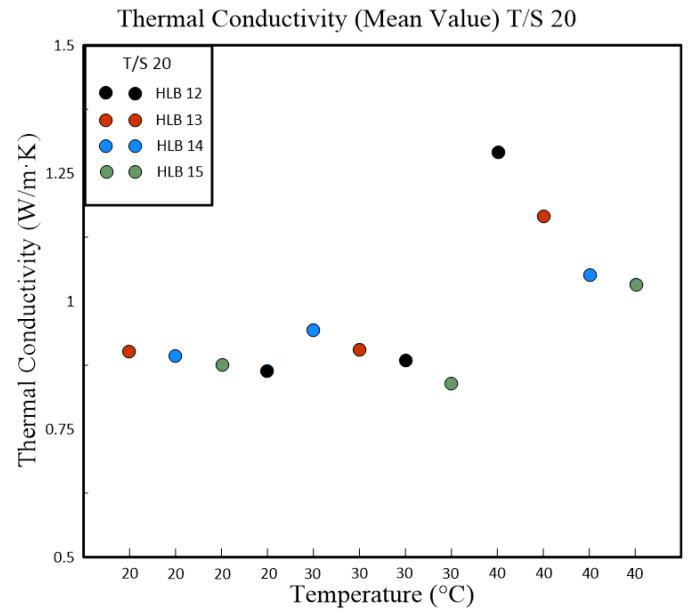
Graph illustration for the mean values of thermal conductivity in relation to the performance (HLB) system [T/S 20].

Table 4 and Table 5 present a detailed analysis of the thermal conductivity properties of nanofluids, which are influenced by their HLB values. These nanofluids were formulated using different polymeric surfactant combinations including Span#20 with Tween#20 (T/S 20) and Span#80 with Tween#80 (T/S 80). The tables also captured the variations in thermal conductivity corresponding to changes in temperature. A clear trend was evident from the data that as the temperature rises, so does the thermal conductivity of the nanofluids. This relationship was particularly pronounced in nanofluids containing T/S 20, which exhibited the best thermal conductivity at an HLB value of 13 when the temperature reached 40 °C. In contrast, nanofluids that incorporate T/S 80 displayed maximum thermal conductivity at a slightly higher HLB value of 14, but at a lower temperature of 30 °C. Figure 6 illustrates the temperature’s impact on the thermal conductivity of nanofluids blended with T/S 20. It highlights a significant sensitivity to temperature changes at an HLB value of 13, where the thermal conductivity increased by 0.2935 W/m∙K for each degree Celsius increase in temperature. It is important to note that the intended duration of the experiment for the T/S 80 HLB group was cut short due to equipment failure. As a result, the data available are limited to the initial five days of the experimental run.

**Figure 7:**
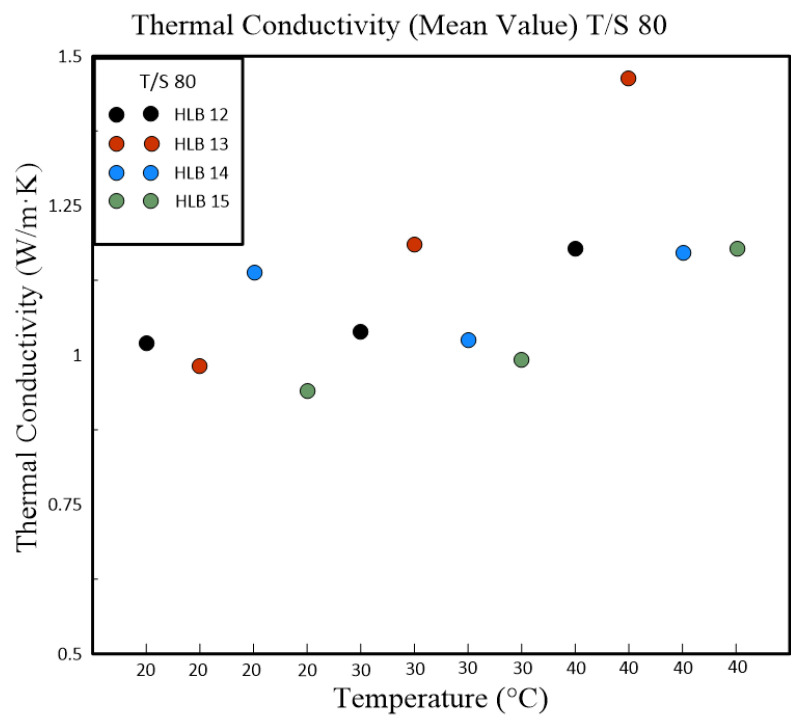
The temperature variation graph based on the thermal conductivity values derived from the HLB values obtained through the combination of Span#80 and Tween#80.

Subsequent experiments, which were conducted with fixed concentrations of powder, polymeric emulsifier, and dispersant, delved into the relationship between nanoparticle size, Zeta potential, and thermal conductivity. The results indicated that the Zeta potential has a numerical effect on the size of the nanoparticle suspension. Moreover, thermal conductivity appeared to be jointly influenced by the size of the nanoparticle suspension and the overall stability of the nanofluid suspension. Our research further revealed a positive correlation between thermal conductivity and temperature across all HLB levels tested. Notably, nanofluids at HLB 12 displayed the highest thermal conductivity at 40 °C. This increase in thermal conductivity is likely attributable to the smaller particle sizes at this HLB level, which enhance heat transfer by providing increased surface area. HLB values of 13 and 14 also registered high thermal conductivity, potentially due to their elevated zeta potentials and improved dispersion stability. The study of T/S 80 mixtures uncovered a consistent rise in thermal conductivity with temperature increments, as depicted in Figure 7. At 40 °C, HLB 13 emerged as the standout, exhibiting the highest thermal conductivity. This observation aligns with zeta potential measurements, where HLB 13 achieved the highest value, suggesting a greater likelihood of achieving stable dispersion. The absence of a similar enhancement in thermal conductivity at HLB 12 within this surfactant group may be attributed to the distinct properties of T/S 80 compared to T/S 20.

## 4. Conclusions

The experimental findings reveal insights into the characteristics of TiO_2_/CuO hybrid nanofluid, highlighting the inverse correlation between particle size, Zeta potential, and thermal conductivity. The analysis led to the following insights, including that utilizing T/S 20 as polymeric emulsifiers resulted in a smaller particle size for the nanofluid suspension. Specifically, the largest particle size, corresponding to an HLB value of 12, was 17% greater than the smallest particle size at an HLB value of 13. On the other hand, the smallest particle size achieved with T/S 80 polymeric emulsifiers, also at an HLB value of 12, showed a mere 5% variance. Regarding Zeta potential, the nanofluid concocted with T/S 80 exhibited a higher negative potential of −30.067 mV, compared to the −25.167 mV observed for the nanofluid with T/S 20.

In comparing thermal conductivities, the nanofluid made with T/S 20 had a 4% lower thermal conductivity than that made with T/S 80. A comprehensive comparison across three parameters revealed that the nanofluid with T/S 80 performed best in two aspects. However, these benefits did not align consistently with the same HLB value. For the nanoparticle suspension size, the nanofluid with T/S 20 at an HLB value of 13 showed the most favorable results. This combination also yielded the best Zeta potential and thermal conductivity, suggesting enhanced stability of the nanoparticle suspension. Therefore, from both economic and environmental perspectives, it is advisable to use T/S 20 polymeric emulsifiers at an HLB value of 13 for nanofluid preparation.

## Figures and Tables

**Table 1:** HLB values of different polymeric emulsifiers.

Properties	Tween 20	Tween 80	Span 20	Span 80
Chemical Formula	C38H113O26	C32H60O10	C18H34O6	C24H44O6
HLB Value	16.7	15	8.6	4.3
Density (Kg/m^3^)	1102	1084	1002	1053
Thermal Conductivity (W/m∙k)	0.18	0.18	0.17	0.17

**Table 2:** Relationship between particle size and varying HBL values over a period of 10 days for tween and span #20.

T/S 20	Particle Size (nm)				
	HLB Value				
Measurements	Temperature	HLB 12	HLB 13	HLB 14	HLB 15
7	20	199.2333	166.5857	173.0857	172.4429
	30	212.3905	177.6381	184.9905	184.1571
	40	215.8333	181.1714	187.981	186.7333
	50	219.481	187.0667	192.9857	186.5048

**Table 3:** Relationship between particle size and varying HBL values over a period of 10 days for tween and span #80.

T/S 80	Particle Size (nm)				
	HLB Value				
Measurements	Temperature	HLB 12	HLB 13	HLB 14	HLB 15
7	20	178.88333	181.01429	183.64286	178.4619
	30	190.41667	192.97619	197.1619	190.3619
	40	194.12143	197.62857	200.48571	194.8857
	50	201.14286	204.39524	208.19524	200.4286

**Table 4:** Temperature variation table based on the thermal conductivity values derived from the HLB values of Span#20 and Tween#20.

T/S 20	Thermal Conductivity (W/m∙K)				
	Temp. (°C)	HLB Value			
Measurements		HLB 12	HLB 13	HLB 14	HLB 15
8 (Mean Value)	20	0.86508	0.90138	0.89363	0.87629
	30	0.88346	0.90592	0.94471	0.83983
	40	1.29133	1.16521	1.05046	1.03396

**Table 5:** Temperature variation table based on the thermal conductivity values derived from the HLB values of Span#80 and Tween#80.

T/S 80	Thermal Conductivity (W/m∙K)				
	Temp. (°C)	HLB Value			
Measurements		HLB 12	HLB 13	HLB 14	HLB 15
4 (Mean Value)	20	1.02033	0.98225	1.13667	0.93927
	30	1.03958	1.18417	1.02342	0.99212
	40	1.17858	1.463	1.17	1.17729

## Data Availability

All data are offered by the authors for reasonable request and the novel core-shell electrodes are available from the authors.
